# Polymorphisms of *CCSER1* Gene and Their Correlation with Milk Quality Traits in Gannan Yak (*Bos grunniens*)

**DOI:** 10.3390/foods12234318

**Published:** 2023-11-29

**Authors:** Guowu Yang, Juanxiang Zhang, Xiaoyong Ma, Rong Ma, Jinwei Shen, Modian Liu, Daoning Yu, Fen Feng, Chun Huang, Xiaoming Ma, Yongfu La, Xian Guo, Ping Yan, Chunnian Liang

**Affiliations:** 1 Key Laboratory of Animal Genetics and Breeding on Tibetan Plateau, Ministry of Agriculture and Rural Affairs, Lanzhou 730050, China; xueshengyangguowu@163.com (G.Y.); juanxiangzhang@163.com (J.Z.); mxy15609445561@163.com (X.M.); marong202017@163.com (R.M.); shenjw9090@163.com (J.S.); liumodian@126.com (M.L.); ydn9907@163.com (D.Y.); feng990111@163.com (F.F.); johnchun825@163.com (C.H.); maxiaoming@caas.cn (X.M.); layongfu@caas.cn (Y.L.); guoxian@caas.cn (X.G.); pingyanlz@163.com (P.Y.); 2 Key Laboratory of Yak Breeding Engineering of Gansu Province, Lanzhou Institute of Husbandry and Pharmaceutical Sciences, Chinese Academy of Agricultural Sciences, Lanzhou 730050, China; 3 College of Life Sciences and Engineering, Northwest Minzu University, Lanzhou 730106, China

**Keywords:** *CCSER1* gene, milk quality, SNPs, Gannan yak

## Abstract

Coiled-coil serine-rich protein 1 (*CCSER 1*) gene is a regulatory protein gene. This gene has been reported to be associated with various economic traits in large mammals in recent years. The aim of this study was to investigate the association between *CCSER1* gene single nucleotide polymorphisms (SNPs) and Gannan yaks and to identify potential molecular marker loci for breeding milk quality in Gannan yaks. We genotyped 172 Gannan yaks using Illumina Yak *c*GPS 7K liquid microarrays and analyzed the correlation between the three SNPs loci of the *CCSER1* gene and the milk qualities of Gannan yaks, including milk fat, protein and casein. It was found that mutations at the g.183,843A>G, g.222,717C>G and g.388,723G>T loci all affected the fat, protein, casein and lactose traits of Gannan yak milk to varying extents, and that the milk quality of individuals with mutant phenotypes was significantly improved. Among them, the milk fat content of AG heterozygous genotype population at g.183,843A>G locus was significantly higher than that of AA and GG genotype populations (*p* < 0.05); the casein and protein content of mutant GG and CG genotype populations at g.222,717C>G locus was significantly higher than that of wild-type CC genotype population (*p* < 0.05); and the g.388,723G>T locus of the casein and protein contents of the mutant TT genotype population were significantly higher (*p* < 0.05) than those of the wild-type GG genotype population. These results provide potential molecular marker sites for Gannan yak breeding.

## 1. Introduction

Yaks (*Bos grunniens*) are mainly distributed in high altitude areas of 2500~6000 m above sea level, and can fully adapt to extremely harsh environments such as those with low temperature, high altitude, and strong ultraviolet radiation [1]. These animals rely entirely on natural grasslands for food and nutrition and do not require supplemental feed [2]. Therefore, yak milk is an exceptionally non-polluting source of green food with great potential for further development and utilization [3]. In the past, Tibetans mainly drank yak milk, known as “liquid gold”. Yak milk and its dairy products provide most of the energy, vitamins and nutrients that Tibetans need [4]. Compared with ordinary milk, yak milk contains higher levels of dry matter, milk fat, milk protein and other nutrients. Among them, yak casein is not only a source of antihypertensive peptides, but also a typical dietary protein, which can be used for various high-value-added functional diets [5]. Also, casein is the foremost source of essential amino acids [6,7]. Yak milk is a valuable source of nutrition and therefore ideal for the production of a wide range of dairy products [8]. In recent years, there has also been a significant increase in interest in yak milk, and more and more research has been conducted on yak milk, such as studies on antioxidant properties [9], lactation mechanisms [10] and yak milk product development [11].

The quantity and quality of milk produced by yaks is regulated by a number of factors, such as seasonal variations, altitude and age. Milk composition is likewise a complex trait influenced by a number of factors, including genetic factors and environmental conditions (such as altitude, temperature, stage of lactation, season, herd and diet) [12,13]. It was found that the regions affecting milk quality traits such as milk protein composition were concentrated on chromosomes 1, 6, 11, 13, 14 and 18 [14]. Various genes have been reported in the literature to be associated with milk production traits, such as *DGAT1*, *HSF1*, *MGST1*, *GHR*, *ABCG2*, *ADCK5* and *CSN1S1* [14,15,16]. The diacylglycerol acyltransferase 1 (*DGAT1*) gene was shown to synergistically affect the milk quality of Egyptian Zaraibi goats through single nucleotide polymorphism (SNP) in the gene [15]. Teng et al. conducted a genome-wide association study of milk production traits in Holstein cows by using medium density microarray data and found that among the new genes that have not yet been reported, coiled-coil serine-rich protein 1 (*CCSER1*) also showed good potential as a candidate gene for milk quality [16]. The *CCSER1* gene, also named *FAM190A* (family with sequence similarity 190, member A), is a regulatory protein gene [17]. As a regulatory or structural component of normal mitosis, when *CCSER1* gene expression is altered, it causes chromosomal instability. In addition, the yak *CCSER1* gene, with a length of 1,597,616 bp and the presence of 10 transcripts, is one of the important functional genes located on chromosome 6 [18]. Studies have found that the *CCSER1* gene is associated with a variety of economic traits, such as growth traits [18,19], feed efficiency [20] and milk quality. Notably, a sequence analysis revealed that the CCSER1 protein has 23 serines in the first 69 amino acids, but no other identified structural domains or known functions [21]. Notably, the *CCSER1* gene has also been studied in cancer therapy. The results of Kang et al. demonstrated that transcript variants of the *CCSER1* gene can serve as clinical therapeutic targets for cancer patients and that the oncogenic properties of the *CCSER1* gene involve in-frame deletions [22].

Low yak milk yield limits the industrial production of yak milk [23]. Therefore, improving milk yield and milk quality are among the important breeding objectives in the yak industry [10]. It has been found that the bioactive components in yak milk have various functional properties, such as antioxidant, anticancer, antibacterial and blood pressure lowering abilities [24]. Compared to the milk fat of other animals, yaks, because of their specific high-altitude, low-oxygen, etc., grazing environments, make it possible for their milk fat to contain certain unique fatty acids [25]. These fatty acids may be associated with potential health advantages, such as anti-cancer and anti-diabetic effects, as well as positive effects on organs such as the brain, heart and eyes [26,27]. Hydrolysates of yak milk casein were found to have the ability to scavenge free radicals such as superoxide and hydrogen peroxide, as well as to inhibit the secretion of inflammatory actives such as pro-inflammatory cytokines and tumor necrosis factor-alpha [28]. Thus, hydrolysates of yak milk casein may be used for the prevention of oxidative-stress- and inflammation-related diseases. It has also been shown that yak casein differs from cow casein in several functional aspects due to its larger particle size, different amino acid sequence and richer mineral concentration, which results in greater conformational stability [29,30]. Therefore, the interest in yak milk quality has increased significantly. Within this is the improvement of milk quality by means of single nucleotide polymorphisms (SNPs), which belong to the third generation of genetic markers [12]. SNPs are changes in the DNA sequence that are prevalent when a base in a gene is altered. This variation mainly includes four types of single gene transition, transversion, deletion and insertion, but transition and transversion are predominant [31]. SNPs have been used to identify genes related to milk production traits in Holstein cows [32], but in dairy cows they have been used mainly for the identification of loci related to milk yield, with fewer studies on specific milk quality. However, SNPs have hardly been studied in yaks for milk yield or milk quality. The Gannan yak is an ancient and primitive livestock breed on the Tibetan Plateau, a unique local genetic resource in Gansu Province, China, and has strong resistance to adversity through long-term natural selection and artificial breeding. It is able to adapt to the ecological conditions of high altitude, strong radiation, a large temperature difference between day and night, a short growing period of pasture grass, extreme cold and little oxygen. In this study, Gannan yak milk was used as the research object. It aimed to investigate the novel SNPs in the *CCSER1* gene and their relationship with the quality of Gannan yak milk.

## 2. Materials and Methods

### 2.1. Ethics Approval

All the animal experiments were approved by the Lanzhou Institute of Husbandry and Pharmaceutical Sciences of the Chinese Academy of Agricultural Sciences (CAAS) with the grant number 1610322020018.

### 2.2. Animal and Milk Composition Analysis

Gannan yak milk samples were collected in July 2023 in Xiahe County, Gannan Tibetan Autonomous Prefecture, Gansu Province (34.99° E, 102.92° N, altitude 3000~3800 m). The Gannan yaks used for sampling were all healthy, disease-free, in the same body condition, had similar milk production, were not artificially supplemented with concentrate and roughage and grazed on natural summer pastures in Gannan, grazing in the same native pasture. A total of 172 yak milk samples were collected, and the parity of lactating yaks was 2–3 times. The collected yak milk was used for milk composition analysis. The analysis included the determination of fat, protein, lactose, casein, non-fat milk solids (SNF), acidity and total solids (TS). A MilkoScanTM FT120 milk composition analyzer (Danish FUCHS Analytical Instruments Ltd., Hellerup, Denmark) was used for determination.

### 2.3. Biological Material Sampling and DNA Extraction

Ear tissue samples from 172 Gannan yaks were collected and stored in liquid nitrogen and brought back to the laboratory. They were preserved at −80 °C until DNA was extracted. Tissue sample DNA was extracted using the magnetic bead method using Magnetic Animal Tissue Genomic DNA Kit (DP341, Tiangen Biochemical Technology Co., Ltd., Beijing, China) [33]. The approximate operation was as follows: take 50 mg of ear tissue, cut it into as small pieces as possible, and add 200 μL of tissue digest GHA and 20 μL of Proteinase K for tissue grinding. Then, 300 uL of lysate GHB was added and incubated at 75 °C for 15 min, after which 350 uL of isopropanol was added. Then, 30 uL of Magnetic Bead Suspension G was added and the liquid was aspirated using the function of magnetic bead adsorption. After that, 700 uL of buffer GDA and 700 uL of rinse solution PWD were added sequentially, and magnetic bead adsorption was performed sequentially to absorb the liquid. The sample was then allowed to dry at room temperature for 10–15 min. Then, 200 uL of elution buffer TB was added and incubated at 56 °C for 10 min. The sample was placed on a magnetic rack for 2 min, and after waiting for complete adsorption of the magnetic beads, the DNA solution was carefully transferred to a new centrifuge tube and stored in appropriate conditions. The concentration of DNA samples was detected by a Qubit fluorescence quantitative instrument. The integrity of the DNA samples was detected with 1% agarose gel electrophoresis.

### 2.4. Genotyping

A total of 172 Gannan yaks were genotyped using an Illumina Yak *c*GPS 7K (Illumina, Huazhi Biotechnology Co., Ltd., Changsha, China) liquid chip. Genotyping was performed using *c*GPS (Genotyping by Pinpoint Sequencing of liquid captured targets). *c*GPS is based on the optimized thermodynamic stability algorithm model to design specific probes for target interval sequences. The synthesized specific probes are used to capture and enrich multiple different target sequences located in different genomic locations through liquid-phase hybridization, and then the library construction and second-generation sequencing are carried out on the target intervals captured and enriched, so as to obtain the genotypes of all SNP/InDel marker sites in the target interval. Fastp was used to control the quality of the raw reads data. Low-quality reads were filtered, and when the base with a mass value of Q ≤ 20 in reads accounted for more than 50% of the total base, the reads pair was removed. Reads with too many N bases were filtered, and reads containing more than 5 Ns were removed, and reads with a length less than 100 were filtered. The genomic location of the SNP was derived from the assembly of the yak reference genome Bosgru v3.0 [34] (GCA_005887515.1). 

### 2.5. SNPs Validation

The genotyping results of the Illumina Yak cGPS 7K microarray were validated by amplifying sequences at the g.183,843A>G, g.222,717C>G and g.388,723G>T loci. PCR amplification primers were designed using Primer Premier 5.0 software (Premier Biosoft International, San Francisco, CA, USA) based on the yak *CCSER1* gene published by Ensemble (accession number: ENSBGRG00000023090). All three loci are located in the yak reference genome Bosgru v3.0 on chromosome 6 of the Yak reference genome (accession number GCA_005887515.1). The g.183,843A>G, g.222,717C>G and g.388,723G>T loci in the Gannan yak were amplified using the primer sequences listed in Table 1. The PCR amplification system was 40 μL: 20 μL of 2× Accurate Taq Master Mix (dye plus), 1 μL of DNA template (100 ng/μL), 1 μL each of the upstream and downstream primers (10 μmol/L), and 17 μL of enzyme-free sterile water. PCR amplification program: pre-denaturation at 94 °C for 30 s; denaturation at 98 °C for 10 s; annealing for 30 s (the annealing temperatures of the three pairs of primers were 57 °C, 50 °C and 53 °C, respectively); extension at 72 °C for 1 min, 35 cycles; extension at 72 °C for 2 min; and storage at 4 °C for cooling. The amplification products were detected with electrophoresis under 1% agarose gel at the end of amplification. Samples that showed specific amplification products of the expected length were sent to Qinke Zexi Biotechnology Co., Ltd. (Xi’an, China) for Sanger sequencing. Sequencing results were analyzed using MEGA11 software (version 11.0). 

### 2.6. Statistical Analysis

Homozygotes (HO) were calculated online using GDICALL (http://www.msrcall.com/gdicall.aspx), (Last accessed on 5 October 2020). The heterozygosity (HE), number of effective alleles (NE), polymorphism (PIC), genotype and allele frequency of the two loci were calculated, and the *p*-values of the chi-square test and Hardy–Weinberg test were calculated. 

We used one-way analysis of variance (ANOVA) in IBM SPSS Statistics 25 (IBM, Armonk, NY, USA) to explore the relationship between *CCSER1* gene polymorphisms and yak milk production traits. In order to analyze the influencing factors of yak milk production traits, we used a general linear model, which was appropriately simplified according to the current situation. The simplified model used Equation (1), where *Yi* is the phenotypic value of milk quality traits, *μ* is the population mean of milk fat traits, *SNPi* is the fixed effect of the genotypic category of the locus, and *e* is the random error effect. Differences between means were tested using Duncan’s multiple comparisons test and results were expressed as mean ± standard deviation. Differences were considered significant at *p* < 0.05.

*Yi* = *µ* + *SNPi* + *e* (1)

## 3. Results

### 3.1. Genotyping Results for CCSER1 and Genetic Parameter Analysis of the Loci in Gannan Yak

**Figure 1:**
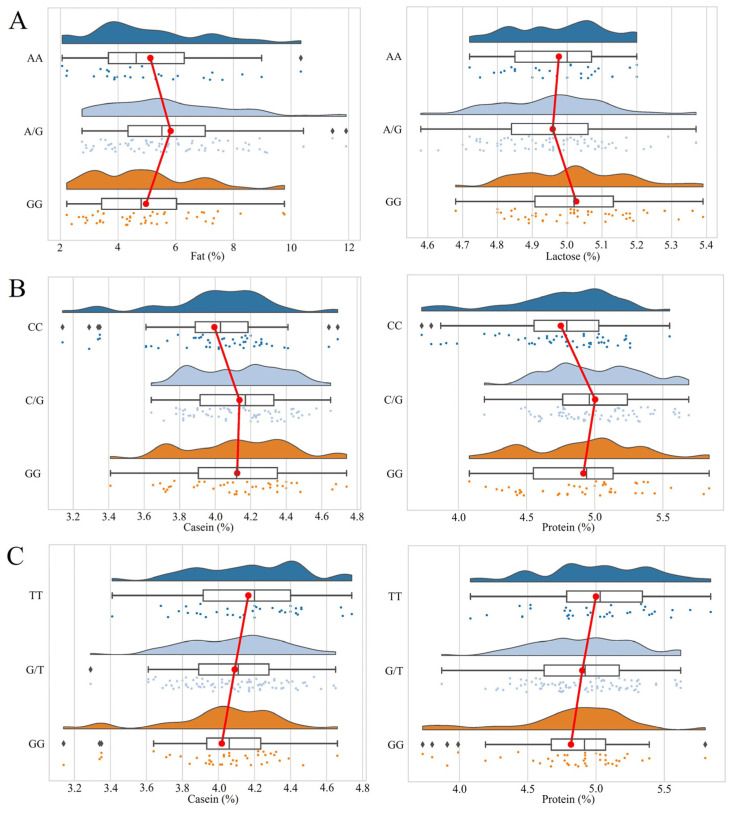
Distribution of all the three genotypes of SNPs (the main significant milk production traits of Gannan yak). (**A**) g.183,843A>G, (**B**) g.222,717C>G, (**C**) g.388,723G>T. The red line represents the mean value of the milk quality trait corresponding to the three genotypes. The three different colored points represent the distribution of data.

**Figure A1:**
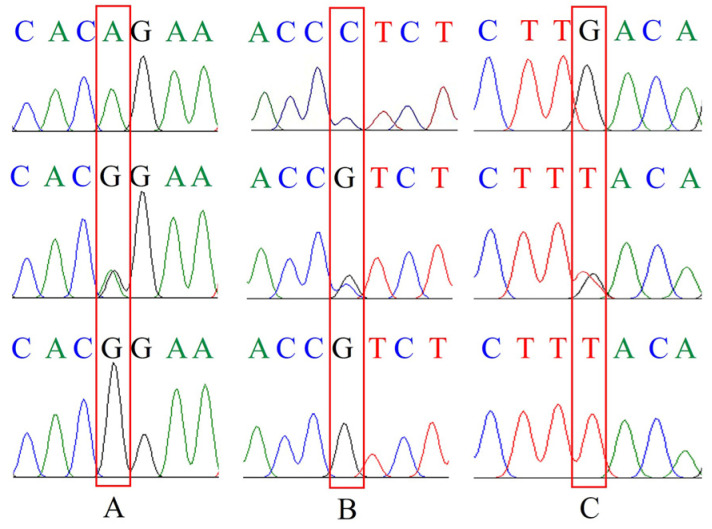
Peak plots of three genotypes at the SNPs locus of the *CCSER1* gene. (**A**) g.183,843A>G. (**B**) g.222,717C>G. (**C**) g.388,723G>T.

The genotype frequency, allele frequency and polymorphism information content of the three SNPs loci of *CCSER1* gene are shown in Table 2. The results showed that the three SNPs loci showed three genotypes in the Gannan yak population. The chip typing results were verified and the results are shown in Figure A1, indicating that the chip typing results are correct. Figure 1 shows the distribution of three genotypes of SNPs in the main milk production traits of the Gannan yak. It can be seen from the figure that the distribution of SNPs is relatively uniform. Among the genotype frequencies of *CCSER1* g.183,843A>G, g.222,717C>G and g.388,723G>T, the genotype frequencies of AG, CG and GT were the highest, and were 0.500, 0.414 and 0.525, respectively. This shows that among the three SNPs sites, heterozygous types are dominant. In g.183,843A>G, the gene frequency of G is 0.571, indicating that the mutant allele accounts for the majority at this site. In g.222,717C>G and g.388,723G>T, the gene frequencies are higher in C and G, respectively, indicating that the unmutated alleles account for the majority in these two loci, but the difference is small. The PICs of g.183,843A>G, g.222,717C>G and g.388,723G>T are 0.370, 0.374 and 0.375, respectively. The polymorphic information contents are between 0.25 and 0.5, and all are moderately polymorphic. Except for the g.222,717C>G site, the other SNPs were consistent with the Hardy–Weinberg equilibrium (*p* > 0.05).

### 3.2. Association Analysis between SNPs Genotypes and Milk Traits in Gannan Yak

The correlation between individual loci and yak milk composition was analyzed based on SNPs genotyping data. The correlation analysis of the g.183,843A>G, g.222,717C>G and g.388,723G>T loci of the *CCSER1* gene in Gannan yaks with milk traits is shown in Table 3. As shown in Table 3, the g.183,843A>G locus was significantly associated with milk fat and lactose traits (*p* < 0.05). The AG heterozygous genotype had significantly higher milk fat content than the AA and GG genotypes (*p* < 0.05). The lactose content of GG pure genotypes was significantly higher (*p* < 0.05) than AG genotypes, but the difference from AA genotypes was not significant (*p* > 0.05). The g.222,717C>G locus was significantly associated with casein, protein, SNF and acidity traits (*p* < 0.05). The casein and protein contents of GG and CG genotypes were not significantly different (*p* > 0.05), but both were significantly higher than the CC genotype (*p* < 0.05). The differences in SNF content and acidity between the mutant genotypes GG and CG were not significant (*p* > 0.05), but the SNF content and acidity of the CG genotype was significantly higher than that of the CC genotype (*p* < 0.05). The g.388,723G>T locus was significantly associated with casein and protein traits (*p* < 0.05). The casein and protein contents were significantly higher in the TT genotype than in the GG genotype (*p* < 0.05), but the difference between the TT and GT genotypes was not significant (*p* > 0.05). This showed that the *CCSER1* gene was mainly associated with the traits of casein, protein, fat and lactose in Gannan yak milk, and both the pure and heterozygous types of the mutant were significantly higher than that of the wild type. This indicated that the individual yak milk quality was higher in the mutant type.

## 4. Discussion

Milk fat is a very high-quality lipid in milk, which is better absorbed by the body because of its high fat-soluble fiber content [8]. It has been found that the digestibility of milk fat in the human gastrointestinal tract is more than 98% [35]. The high protein level in milk can provide essential amino acids for the growth of newborns, and some special proteins can also improve immunity and promote the utilization of trace elements [36]. Moreover, yak milk is rich in calcium, protein, peptides, amino acids, iron, phosphorus, vitamins and other nutrients and functional substances, and yak milk has a higher proportion of iron, zinc, manganese, selenium, lactoferrin, immunoglobulin, etc., compared with ordinary cow’s milk [9]. In terms of safety and hypoallergenicity, yak milk is closer to breast milk than ordinary cow’s milk and goat’s milk, and has the effect of lowering blood sugar, promoting growth and development, providing anti-inflammation properties and strengthening immunity [37,38]. Therefore, yak milk has higher nutritional characteristics, with fat and protein content being important indicators of quality in yak milk. Similarly, the intake of protein and fat plays a very important role in the growth and development of mammals. The results of this experiment showed that the g.183,843A>G locus mainly affected the milk fat and lactose of Gannan yaks, in which the AG heterozygous group had significantly higher milk fat content than the two pure groups. However, g.222,717C>G and g.388,723G>T mainly improved the milk protein of the Gannan yak, and both mutant heterozygous populations had significantly higher protein content than the wild-type population. The experimental results indicated that the mutation of SNPs significantly improved the milk quality of the Gannan yak.

SNPs are an important basis for the study of genetic variation in livestock and poultry. SNPs have the characteristics of high reliability, wide distribution and ease of analysis [39]. They play an important role in analyzing the genetic basis of important economic traits of livestock and poultry. In this study, we found that when the base of the *CCSER1* g.183,843A>G locus was mutated from A to G, the mutant genotypes had a significant effect on fat and lactose in Gannan yaks on analyzing the correlation between the loci of the *CCSER1* gene and milk traits. When the base of the *CCSER1* g.222,717C>G locus was mutated from C to G, both heterozygous and pure genotypes of the mutation had a significant improvement on casein, protein and SNF in Gannan yaks. The mutation of the *CCSER1* g.388,723G>T locus was basically the same as that of the *CCSER1* g.222,717C>G locus, and the mutant genotype TT also had a significant improvement on casein and protein and SNF in Gannan yaks, with significant improvement in casein and protein. Overall, the mutations of all three SNPs positively affected the milk quality traits of Gannan yaks to different degrees. Clancey et al. detected SNPs and gene sets associated with 305 standard milk yield in 781 primiparous Holstein cows, and identified new candidate genes that cause milk production variation [32]. Jiang et al. analyzed the SNPs of 480 yaks using a commercial high-density (600 K) yak SNP chip, and identified 12 and 4 SNPs that may be related to the weight of male and female yaks, respectively [40]. Nevertheless, the study of yak SNPs is still in its infancy, and a large amount of experimental data are needed to elucidate the potential relationship between SNPs and economic traits.

It was found that *CCSER1* is localized to the γ-microtubulin ring complex in early mitosis, as well as to an intermediate in late cytokinesis [21]. Therefore, it has been concluded that the *CCSER1* gene is an important regulator or structural molecule that plays an important regulatory role in normal cellular mitosis [16,21], which may alter chromosome stability and somatic cell division. Based on bovine gene expression profiles, it is expressed in 85 tissues or cell types in cattle, while its expression is relatively high in the mammary gland [41]. The results of the present study also confirmed that the *CCSER1* gene had a significant effect on milk quality traits in the yak population. This may provide new insights to improve yak milk quality traits through selection strategies.

Here, we identified three SNPs loci, g.183,843A>G, g.222,717C>G and g.388,723G>T, in the *CCSER1* gene. Among them, the g.183,843A>G locus is located in the coding region of the *CCSER1* gene, which has an impact on gene expression and function, and thus may have a more direct effect on milk quality. In addition, we found that the g.222,717C>G and g.388,723G>T loci are located in the intronic region of the *CCSER1* gene, which does not affect their association with yak milk quality. Although introns are sequences with no coding function in genes, they have important roles in the regulation of gene expression [42], and the important function of SNPs in introns in altering the transcription level of genes has been elucidated [43]. They cause shear abnormalities by altering the structure of the shear site, which may lead to changes in protein structure and function [44]. In addition, several SNPs sites in the intron region of the erb-b2 receptor tyrosine kinase 2 (*ERBB2*) gene were reported to be significantly associated with milk protein content in Chinese Holstein cows [45]. Five SNPs (g.29029T>C, g.29050A>G, g.29245C>T, g.29305C>T and g.29347T>C) in the intron region of sorbin and the SH3 domain-containing 1 (*SORBS1*) gene were reported to be significantly associated with milk fat traits in cattleyak [46]. All of the above studies had results similar to this study. Therefore, the role of mutations in the g.222,717C>G and g.388,723G>T loci of the *CCSER1* gene in Gannan yaks on the genetic characterization of this gene still needs to be further confirmed. The polymorphisms of *CCSER1* g.183,843A>G, g.222,717C>G and g.388,723G>T were 0.370, 0.374 and 0.375, respectively, and the polymorphic information content of the three SNPs was moderate. Among them, the *p*-value of the *CCSER1* g.222,717C>G locus was greater than 0.05, indicating that the population at this locus was genetically unbalanced and deviated from Hardy–Weinberg. This may be due to a variety of factors such as sampling bias, natural selection or human intervention. In our study, we used liquid-phase microarray genotyping, which is a technique for precise positional sequencing typing based on the liquid-phase capture of target sequences and is specifically designed for genotyping large samples at specific SNP loci. Therefore, genotyping is unlikely to be the cause of this phenomenon.

Consistent with the present study, the functional role of the *CCSER1* gene for milk production traits was also identified as a positional candidate gene for lactation persistence in Holstein cattle. Thus, the results of the present study and previously published studies suggest that *CCSER1* is a promising candidate gene with strong genetic effects on milk production traits in Gannon yaks. In addition, single nucleotide polymorphism-based and correlation analyses also suggest that the novel SNPs in the *CCSER1* gene may be used as potential genetic markers for genetic improvement in yak breeding programs.

## 5. Conclusions

In this study, we explored the *CCSER1* gene polymorphisms, identified three SNPs loci and analyzed the relationship between these polymorphisms and the milk quality of Gannan yaks. The results showed that all three SNPs of the *CCSER1* gene were moderately polymorphic. Correlation analysis revealed that the mutant genotype of the *CCSER1* g.183,843A>G locus in Gannan yaks resulted in a significant (*p* < 0.05) increase in milk fat content, whereas the mutant genotypes of both *CCSER1* g.222,717C>G and g.388,723G>T loci resulted in a significant increase (*p* < 0.05) in the casein and protein content of Gannan yak milk. Therefore, mutations in the *CCSER1* g.183,843A>G, g.222,717C>G and g.388,723G>T loci all significantly improved the quality traits of Gannan yak milk. The identification of these SNPs opens the way for further research and applications in the selective breeding of Gannan yaks. The results of this study can help to develop and optimize the milk quality of Gannan yaks.

## Figures and Tables

**Table 1:** SNPs amplification primer sequence of *CCSER1* gene.

SNPs	Primer Sequence (5′–3′)	Product Size
g.183,843A>G	F: TAACAGAACGGGCAGGTAGC	633 bp
	R: AAATCAGCATACCTTTGGCAGG	
g.222,717C>G	F: AATAAATGATGTCGCCAATA	317 bp
	R: CTGCGTAGAATACAAAAGAAT	
g.388,723G>T	F: AGCACCTTCTTCTTACTCAT	404 bp
	R: ATTGTTCTGCTGCTGGGATT	

**Table 2:** Variation information and diversity parameters of *CCSER1* loci.

SNPs	Position	Genotypic Frequencies			Allelic Frequencies		He	Ne	PIC	*p* Value
g.183,843A>G	Exon	AA	AG	GG	A	G	0.490	1.960	0.370	0.793
		0.179	0.500	0.321	0.429	0.571				
g.222,717C>G	Intron	CC	CG	GG	C	G	0.498	1.994	0.374	0.030
		0.321	0.414	0.265	0.528	0.472				
g.388,723G>T	Intron	GG	GT	TT	G	T	0.499	1.996	0.375	0.513
		0.259	0.525	0.216	0.522	0.478				

Note: He: heterozygosity; Ne: effective number of alleles; PIC: polymorphism. PIC < 0.25, low polymorphism; 0.25 < PIC < 0.5, moderate polymorphism; PIC > 0.5, high polymorphism; *p* > 0.05 suggests that the population gene is in the Hardy–Weinberg balance and the sample comes from the same Mendel population.

**Table 3:** Correlation analysis between *CCSER1* g.183,843A>G, g.222,717C>G, g.388,723G>T and milk traits in Gannan yak.

SNPs g.183,843A>G							
Genotype	Casein/%	Protein/%	Fat/%	SNF/%	Lactose/%	Acidity/° T	TS/%
AA	4.10 ± 0.25	4.92 ± 0.34	5.12 ± 2.09 ^b^	11.27 ± 0.38	4.97 ± 0.14 ^ab^	12.44 ± 1.15	16.57 ± 2.58
AG	4.11 ± 0.28	4.93 ± 0.38	5.82 ± 2.06 ^a^	11.28 ± 0.46	4.96 ± 0.16 ^b^	12.53 ± 1.25	16.90 ± 2.43
GG	4.05 ± 0.33	4.83 ± 0.45	4.97 ± 1.84 ^b^	11.23 ± 0.55	5.03 ± 0.16 ^a^	12.15 ± 1.41	16.49 ± 2.85
*p*-Value	*p* = 0.453	*p* = 0.351	*p* = 0.042	*p* = 0.826	*p* = 0.047	*p* = 0.268	*p* = 0.652
**SNPs g.222,717C>G**							
**Genotype**	**Casein/%**	**Protein/%**	**Fat/%**	**SNF/%**	**Lactose/%**	**Acidity/° T**	**TS/%**
CC	3.99 ± 0.32 ^b^	4.75 ± 0.41 ^b^	5.12 ± 1.97	11.13 ± 0.49 ^b^	5.02 ± 0.15	12.01 ± 1.18 ^b^	16.77 ± 2.86
CG	4.14 ± 0.24 ^a^	5.00 ± 0.34 ^a^	5.26 ± 2.14	11.37 ± 0.38 ^a^	4.96 ± 0.16	12.71 ± 1.14 ^a^	16.49 ± 2.09
GG	4.12 ± 0.30 ^a^	4.92 ± 0.42 ^a^	5.98 ± 2.53	11.25 ± 0.54 ^ab^	4.98 ± 0.16	12.33 ± 1.50 ^ab^	16.99 ± 2.95
*p*-Value	*p* = 0.020	*p* = 0.002	*p* = 0.069	*p* = 0.024	*p* = 0.083	*p* = 0.012	*p* = 0.607
**SNPs g.388,723G>T**							
**Genotype**	**Casein/%**	**Protein/%**	**Fat/%**	**SNF/%**	**Lactose/%**	**Acidity/° T**	**TS/%**
GG	4.02 ± 0.32 ^b^	4.82 ± 0.43 ^b^	5.37 ± 1.97	11.25 ± 0.47	5.03 ± 0.17	12.20 ± 1.25	16.31 ± 2.09
GT	4.09 ± 0.27 ^ab^	4.90 ± 0.36 ^ab^	5.51 ± 2.09	11.24 ± 0.47	4.98 ± 0.15	12.39 ± 1.26	16.78 ± 2.68
TT	4.17 ± 0.31 ^a^	5.00 ± 0.42 ^a^	5.28 ± 2.03	11.32 ± 0.49	4.94 ± 0.14	12.61 ± 1.40	17.00 ± 2.90
*p*-Value	*p* = 0.038	*p* = 0.043	*p* = 0.852	*p* = 0.761	*p* = 0.582	*p* = 0.375	*p* = 0.406

Note: In the same group of data, different lowercase letters showed significant differences (*p* < 0.05). Data are presented as the mean ± standard deviation.

## Data Availability

The data presented in this study are available on request from the corresponding author.
